# POCUS in Intensive Care Nephrology

**DOI:** 10.24908/pocus.v7iKidney.15016

**Published:** 2022-02-01

**Authors:** Randi Connor­-Schuler, Jonathan Suarez

**Affiliations:** 1 Division of Pulmonary, Allergy, Critical Care and Sleep Medicine, Department of Medicine, Emory University Atlanta, Georgia; 2 Division of Nephrology, Department of Medicine, Emory University Atlanta, Georgia

**Keywords:** Nephrology, POCUS, Ultrasound, Intensive care, ICU, acute kidney injury, AKI

## Abstract

Acute kidney injury (AKI) is a significant problem for patients admitted to the intensive care unit (ICU), both due to the high incidence and associated mortality with rates of AKI requiring renal replacement therapy (RRT) of over 5%, and mortality rates with AKI of over 60% 1, 2.Ultrasound can be used to identify those at risk for AKI and assist with AKI management. Risk factors for AKI in the ICU not only include hypoperfusion but also venous congestion and volume overload. Volume overload and vascular congestion are associated with multi-organ dysfunction and worse renal outcomes. Daily and overall fluid balance, daily weights, and physical examination for edema can be inaccurate and belie true systemic venous pressure 3, 4, 5. Bedside ultrasound allows providers to evaluate vascular flow patterns and obtain a more reliable evaluation of volume status to guide and individualize therapies. Cardiac, lung, and vascular patterns on ultrasound can identify preload responsiveness, which should be assessed to safely manage ongoing fluid resuscitation and assess for signs of fluid intolerance. Here we present an overview in the use of point of care ultrasound with particular emphasis on nephro-centric strategies, namely in the identification of the type of renal injury, renal vascular flow assessment, the static measure of volume status, as well as dynamic evaluation for volume optimization in critically ill patients.

## Diagnosis and management of acute kidney injury using point of care ultrasound (POCUS)

Acute kidney injury is a pervasive issue for patients admitted to the intensive care unit (ICU). Not only is it common, but it is also associated with high mortality rates of up to 60%, so early recognition and optimization of renal resuscitation is paramount 1, 2. Bedside ultrasound assists with determining the etiology for AKI and allows providers to evaluate vascular flow patterns to obtain a reliable evaluation of volume status and guide individualized therapies to help improve renal outcomes.

### Hemodynamic AKI

**Figure 1:**
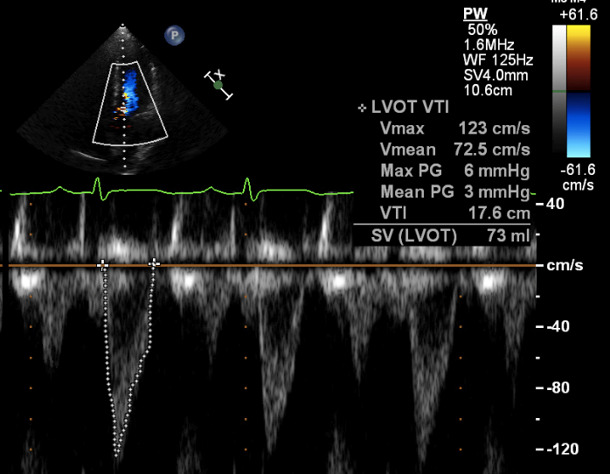
LVOT VTI calculated by obtaining a 5-chamber apical view and using a pulsed-wave Doppler at the opening of aortic valve to trace along the edge of the velocity to measure the area under the curve.

Hemodynamic AKI involves hypoperfusion and is attributable to hypovolemia or low oncotic states seen in heart or liver failure. Cardiac ultrasound can be highly beneficial to help further delineate between these potential etiologies. The ejection fraction depends on ventriculo-arterial coupling, thus is determined by both intrinsic cardiac function and the afterload state 6. As such, hyperdynamism, or an ejection fraction >70%, may be seen either in states of hypovolemia or vasoplegia as seen in liver failure or sepsis. Heart failure on the other hand is not typically associated with a hyperdynamic heart 7. If hyperdynamism is visualized, performing a left ventricular outflow tract (LVOT) velocity time integral (VTI) measurement can help distinguish between hypovolemia and vasoplegia. A low VTI, or one <18 cm, is consistent with low stroke volume and low cardiac output, as seen in hypovolemia. Conversely, a high VTI, or >22cm, is more consistent with a high output state as seen with vasoplegia and distributive shock (Figure 1) 8. 

### Intrinsic AKI

Renal Resistive Index

The renal resistive index (RRI) evaluates the resistance to flow and as such can be used to assess macrovascular perfusion to the kidney. The RRI is obtained at the level of the segmental arteries and is calculated as follows 9:

RRI = [(peak systolic velocity – end diastolic velocity)/peak systolic velocity] 

**Figure 2:**
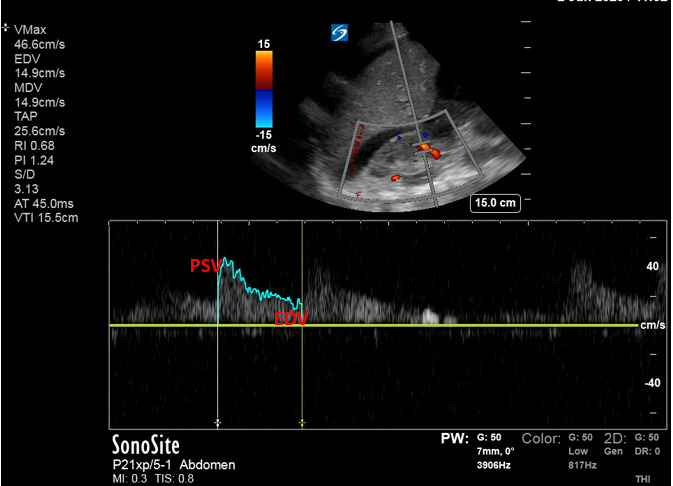
Renal Resistive Index (RRI) can be calculated automatically by ultrasound or plugging into formula: RRI = [(PSV– EDV)/PSV], where PSV = peak systolic velocity, EDV = end diastolic velocity.

Normal values are roughly 0.6 with levels >0.70 used as a cut-off for an abnormal value in native kidneys and 0.8 in transplanted kidneys (Figure 2). Higher levels portend to worse renal outcomes and are predictive for AKI in general as well as for severity of AKI in various patient populations 9, 10, 11, 12. 

The RRI may be increased in CKD or AKI when due to obstruction, acute rejection, hepatorenal syndrome, or acute tubular necrosis (ATN) 9. The RRI can be helpful to distinguish between AKI secondary to pre-renal azotemia, where it remains normal, compared to ATN when it is often elevated 13. Studies have shown a resistive index >0.75 correlates to a diagnosis of ATN with over 90% of patients having ATN compared to only 20% with pre-renal azotemia, most of which had hepatorenal syndrome 9, 12, 13, 14, 15, 16.

The RRI has several limitations. Firstly, it can be affected by heart rate as tachycardia can falsely elevate the end diastolic pressure and thus falsely decrease the RRI 17. The RRI may also be affected by increased intraabdominal pressure, medications, and the location where it is measured 18, 19. Previous studies have shown reproducibility of RRI among different operators, however most studies involved expert sonographers and intra-observer variability may exist in novice sonographers 20, 21.Lastly, while the RRI evaluates macrovascular perfusion, it is unable to assess slow flow velocities in vessels <2 mm and microcirculatory perfusion plays an important role in AKI due to sepsis 22, 23. 

### Post-renal AKI

Hydronephrosis

**Figure 3:**
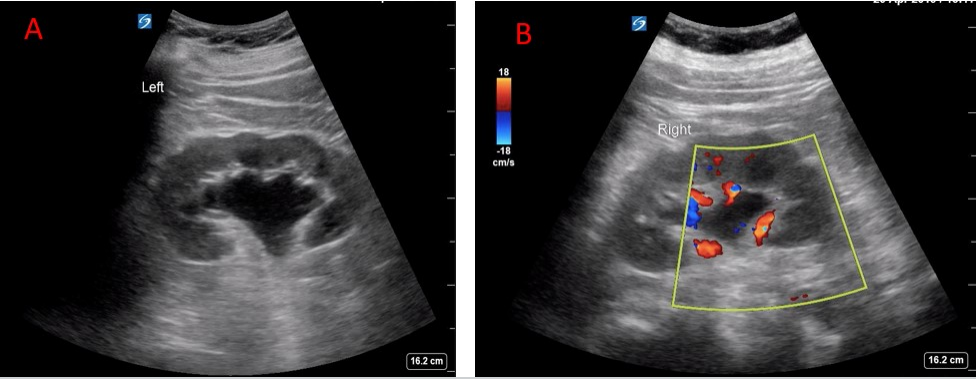
A) Example of moderate hydronephrosis, B) Example of hydronephrosis with color to rule out vasculature.

Ultrasound is the main imaging modality to evaluate for hydronephrosis, which causes 1-3% of AKI in the ICU and is seen on ultrasound in up to 95% of patients with obstruction^24^, ^25^, ^26^, ^27^. There are different degrees of hydronephrosis including mild, moderate, and severe with varying degrees of disruption of the pelvicalyceal system (Figure 3) 10, 28. Of note, it is critical to place color Doppler over the kidney as renal vasculature or vascular malformations can easily mimic hydronephrosis (Figure 3). Although AKI in the setting of obstructive uropathy in the ICU is rare, it is a potentially reversible cause of AKI that can change your management. Ultrasound is a vital tool to make this diagnosis quickly at the bedside that should be employed by every provider for any patient presenting with AKI.

## Nephroprotective strategies

While it is important to correct hypovolemia and supplement preload during early management, particularly for distributive and hypovolemic shock, guiding ongoing fluid administration after the initial resuscitation can be nebulous and nuanced. It has been proposed by Malbrain et al. that there are several stages of fluid management, or the 4 D’s. These include drug, dosing, and duration which revolve around the type and amount of fluid resuscitation as well as de-escalation as it relates to fluid removal 28. POCUS can be used during both the resuscitative and de-resuscitative stages of management. 

### The use of POCUS to optimize resuscitation

Static markers such as CVP were used in the past, however, have not been shown to reliably identify patients that benefit from the administration of fluids 29. As such, there is an emphasis on using dynamic measures to assess for fluid responsiveness, which is defined as an increase in cardiac output or cardiac index by 15% or more in response to a fluid challenge. Many of these dynamic measures used to evaluate for fluid responsiveness rely on heart-lung interactions during mechanical ventilation and involve the use of ultrasound. It is important to note limitations for the following assessments of fluid responsiveness as they can be affected by spontaneous breathing, arrhythmias, or low tidal volume ventilation 30, 31, 32, 33, 34, 35. 

### Venous System – Inferior Vena Cava

**Figure 4:**
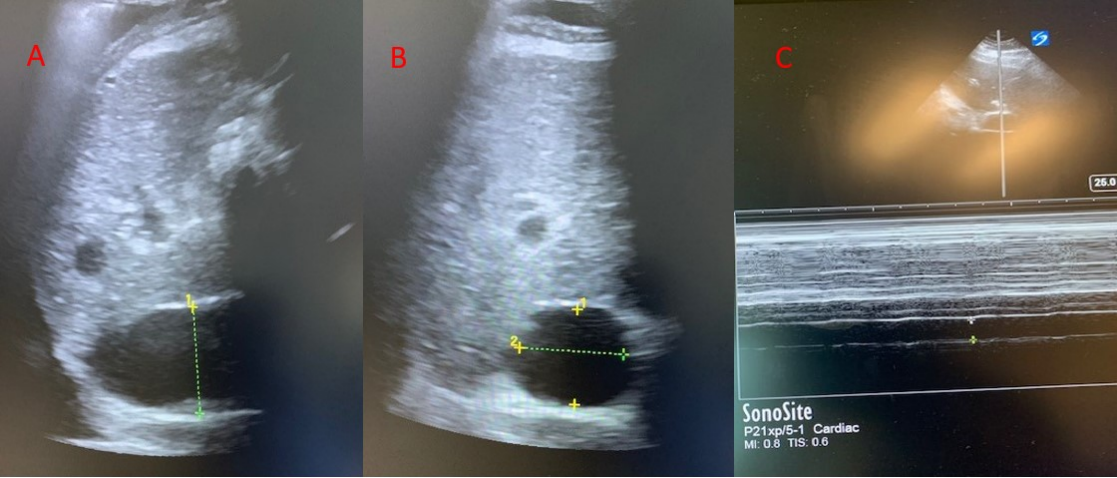
A) IVC-long axis view, B) IVC-short axis view, C) IVC- M-mode to assess for respiratory variation and collapsibility.

The inferior vena cava (IVC) should be viewed in long axis from the subcostal position with measurements taken 2 cm distal to the junction with the right atrium, or just distal to the inlet of the hepatic vein. Due to differences in IVC collapsibility by sampling location and displacement associated with respirophasic cycles multiple measurements at different levels should be taken, including the short axis (Figure 4) 36, 37. There have been many studies looking at the IVC for fluid responsiveness and the best evidence exists for patients on mechanical ventilation 38, 39. IVC collapsibility, when used in spontaneously breathing patients, loosely correlates to CVP and acts as a static measure of volume status. Conversely, given the vessel distends on receiving a mechanical breath instead of collapsing, IVC distensibility assesses vessel compliance in addition to right atrial pressure and the transmural gradient between intraabdominal and intrathoracic pressures. The IVC distensibility thus provides a better assessment for volume responsiveness from a physiologic perspective for patients on mechanical ventilation compared to those with spontaneous respirations, and multiple studies support this physiologic rationale 40. 

Measurement of IVC size and its collapsibility can act as a surrogate measure for CVP and RAP where a size >2.1 cm with <50% collapsibility indicates a CVP of 15. Conversely, an IVC <2.1 cm with >50% collapsibility is consistent with a normal RAP of 3 mmHg 40. An underfilled IVC can help identify hypovolemia, particularly in patients with known volume losses. In states such as sepsis or liver failure, however, where vasoplegia occurs and there is loss of vascular tone, care should be taken to use measures other than the IVC to determine whether to give additional IVF. In such patients, there is a higher ratio of unstressed to stressed volume, and volume overload would need to occur to manifest any change in the stressed volume as visualized by IVC measurement. Conversely, a plethoric IVC indicates the presence of hypervolemia and can help rule out hypovolemic shock. However, the IVC may be plethoric without the presence of hypervolemia and volume overload when tricuspid regurgitation, elevated right sided heart pressures from elevated pulmonary pressures, and cardiac tamponade are present. 

IVC size and collapsibility in spontaneously breathing patients can be affected by elevated intra-abdominal pressures and heavy spontaneous breathing efforts and it is only loosely related to CVP. Careful consideration should be taken when using ultrasound of the IVC in isolation to determine volume status. Rather, it should be used in conjunction with the overall clinical context and constellation of ultrasound findings.

### The use of POCUS to optimize de-resuscitation

It is critical to avoid volume overload which causes tissue edema and prolonged ventilator weaning, and is an independent predictor of mortality in ICU patients, particularly those with sepsis, ARDS, intra-abdominal hypertension, and acute kidney injury. As such, de-resuscitation is a critical phase of a patient’s ICU management 41, 42, 43, 44, 45. POCUS can be used to guide this phase of therapy. 

### Cardiac Ultrasound

In addition to assessing the left ventricle (LV), right ventricular size and function should be evaluated, particularly with regards to assessing volume status. Enlargement of the right ventricle (RV) can be either a sign of volume or pressure overload. The parasternal short axis view can be utilized to evaluate which of these processes predominates as septal bowing seen in systole corresponds to pressure overload on the RV, suggesting an etiology such as pulmonary embolism, whereas septal bowing in diastole corresponds to volume overload that should prompt diuresis 46, 47.

Diastolic function can also be used to assess volume status where e’ maximal velocity reflects the rate of LV relaxation and E/e’ correlates to LV filling pressures. There are studies in the ICU showing an abbreviated assessment can be valuable and diagnostic for increased left ventricular end-diastolic pressure (LVEDP) 48, 49.

**Figure 5:**
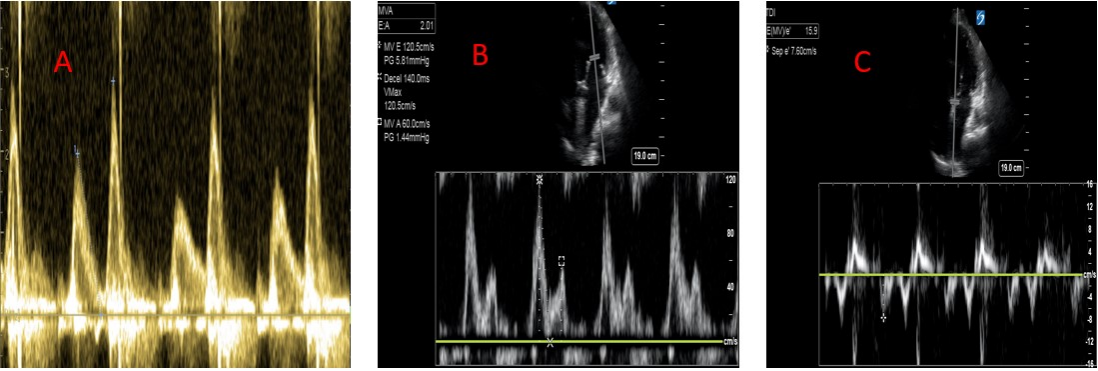
A) Grade 1 diastolic dysfunction showing E<A wave, B) Grade 3 diastolic dysfunction showing E/A wave >2, C) E/e’ > 15 consistent with raised LVEDP.

Using pulse-wave Doppler with the gate just above the mitral valve leaflet tips, obtain a spectral waveform. An E < A wave indicates grade 1 diastolic dysfunction (DD) with normal left atrial pressure and thus normal LVEDP (Figure 5). If E > A, perform an e’ measurement placing TDI over the septal and/or lateral mitral annulus (Figure 5). An E/e’ >15 is consistent with an elevated LVEDP that should prompt diuresis 50, 51.Studies have shown an E/e’ >15 in the point of care setting to be 100% sensitive and 95% specific for heart failure when found in conjunction with B lines on lung ultrasound, also performing better than other modalities such as BNP and chest x-ray for diagnosing decompensated heart failure 50. Conversely, if the E/e’ is <8, then the LVEDP is likely normal 51.

### Lung Ultrasound

Depending on one’s comfort level with more advanced echocardiography techniques, a lung ultrasound is simple to perform and can also be beneficial in assessing volume status in conjunction with other modalities. The presence of A-lines indicates normal lung aeration with dry interlobular septa and low or normal LAP 52. A B-profile, on the other hand, indicates an underlying process is present causing increased density to lung tissue consistent with alveolar-interstitial syndrome 53, 54. When present bilaterally and diffusely, typically this indicates the presence of pulmonary edema, a diagnosis heightened by the concomitant presence of pleural effusions. The presence and quantity of B lines from suspected pulmonary edema correlates well with an elevated LVEDP 55. Ultrasound is more sensitive for both pulmonary edema and pleural effusions compared to chest radiography, and is rapidly performed at the bedside 53, 54. However, lung ultrasound also has its limitations as the presence the presence of B-lines does not always correlate with increased extra-vascular lung water and could be related to pneumonia, ARDS, or pulmonary fibrosis. 

### Abdominal Ultrasound – Venous System

It is critical to identify the presence of volume overload and venous congestion not only in patients with heart failure or cardiogenic shock, but in all forms of pathology. As volume reaches the upper limits of systemic venous capacitance, venous pressure will rise causing congestive changes that can be visualized on ultrasound of the IVC, portal vein, hepatic veins, and the interlobar renal veins 56, 57, 58, 59.

Hepatic Vein

**Figure 6:**
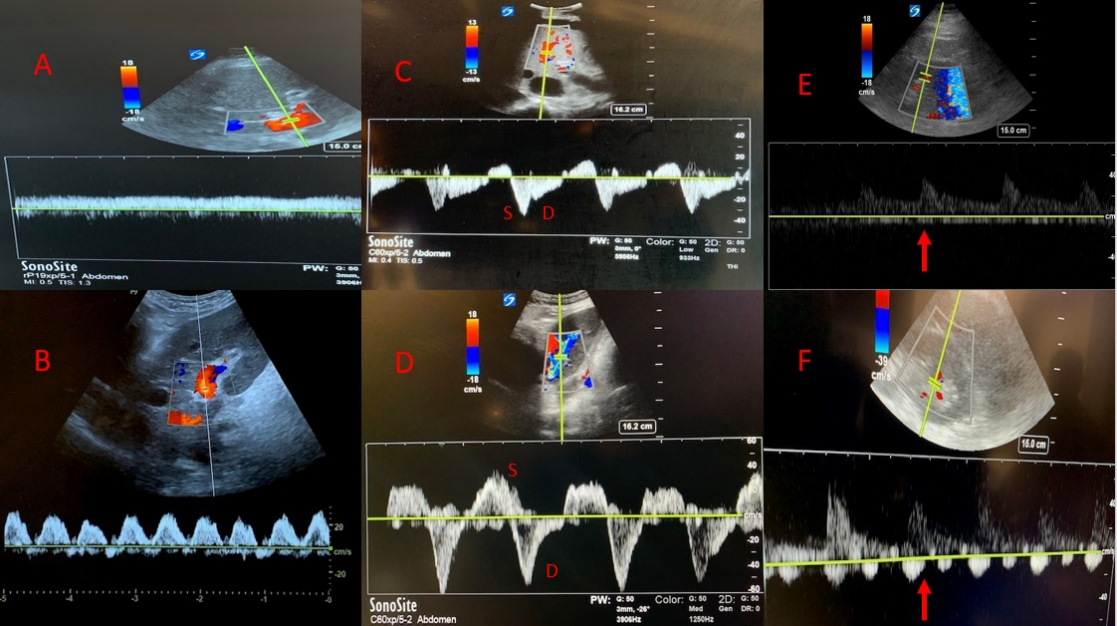
A) Portal vein doppler showing a continuous flow pattern consistent with a normal RAP, B) Portal vein doppler showing biphasic flow consistent with elevated RAP, C) Hepatic vein Doppler showing anterograde S and D wave consistent with a normal RAP, D) Hepatic vein Doppler showing reversal of flow with retrograde S wave consistent with an elevated RAP, E) Renal vein Doppler showing continuous flow consistent with a normal RAP, F) Renal vein Doppler showing discontinuous flow consistent with an elevated RAP.

Given the multivariate etiologies for its morphology and respirophasic behavior, other venous waveforms should be assessed in conjunction with the IVC to identify venous hypertension and congestion. The hepatic vein will be thin-walled and branch off of the IVC. It can be evaluated from the right coronal plane in long axis view with the pulsed-wave Doppler gate placed perpendicular to the flow to obtain a waveform. Alternatively, the hepatic vein can be obtained anteriorly with the IVC in short axis. In the absence of pathology, there should be an anterograde S and D wave (Figure 6). There is an initial retrograde a wave generated by increased RAP from atrial contraction at end diastole, which is followed by an anterograde S wave caused by decreasing RAP during early mid-systole. If the tricuspid valve is open, however, retrograde deflection can occur causing reversal of the S wave, as seen with tricuspid regurgitation or significant venous congestion. The final D wave is an anterograde deflection caused by decreasing RAP from rapid early diastolic right ventricular filling. With increasing venous congestion, there is a spectrum of waveform changes that occur. The S wave is typically larger, however, with increased RAP and mild congestion, the D wave becomes deeper than the S wave, and with severe congestion, there will be reversal of flow with a retrograde S wave (Figure 6). 

### Portal Vein

In contrast to the hepatic veins, the portal vein and its branches will have thicker, echogenic walls and will have flow directed towards the transducer. The portal vein should be assessed for pulsatility. Normally the portal vein should have only mild respirophasic variation and should be fairly uniform. With increasing congestion and right atrial pressure elevation, however, the cyclical pressure changes in the right atrium from cardiac contractility get transmitted to the portal circulation creating a pulsatile flow pattern. Once severe congestion is present, the flow form may become biphasic with a sinusoidal retrograde and anterograde flow pattern (Figure 6). The pulsatility index can be measured as follows where normal PI is less than 30% and significant venous congestion is present with a PI >50%: 

PI (%) = 100 x [(maximum velocity – minimum velocity)/maximum velocity]

There have been several studies looking at portal vein pulsatility in patients with congestive heart failure and following cardiac surgery. In one study, of the patients who had portal flow pulsatility, over 60% developed an AKI in the week following surgery 58.

### Interlobar Renal Veins

The interlobar renal veins can also be assessed for the presence of venous congestion. Studies looking at ultrasound findings of venous congestion in patients with congestive heart failure have shown that portal vein pulsatility was also associated with alterations of intrarenal venous flow 58, 59.

After obtaining a view of the kidney, color Doppler should be placed over the corticomedullary junction to pick up flow through the interlobar vessels with the pulsed-wave Doppler gate placed at the corticomedullary junction ideally obtaining both arterial and venous waveforms. The resistive index can be calculated from the arterial waveform and the venous waveform morphology can also be assessed. Venous flow should be monophasic and continuous, however, with increasing congestion will become pulsatile throughout the cardiac cycle, and with severe congestion venous flow will only be present in diastole (Figure 6).

## Conclusion

Bedside ultrasound can be used to help delineate etiologies for acute kidney injury and to assist in management. Furthermore, ultrasound should be used daily in the ICU to help prevent renal injury by optimizing volume management throughout a patient’s admission. Ultrasound should also be used not only in the initial resuscitation, but to guide de-resuscitation as well. More studies are needed regarding the use of bedside ultrasound to better optimize renal perfusion as well as to systematically guide volume removal by continuous renal replacement therapy.

## Disclosures

The authors have no conflicts of interest to declare.

## References

[R167200426977102] Uchino S, Kellum J A, Bellomo R, Doig G S, Morimatsu H, Morgera S, Schetz M, Tan I, Bouman C, Macedo E, Gibney N, Tolwani A, Ronco C (2005). Beginning and Ending Supportive Therapy for the Kidney (BEST Kidney) Investigators. Acute renal failure in critically ill patients: a multinational, multicenter study. JAMA.

[R167200426977132] Schneider A, Johnson L, Goodwin M (2011). Bench-to-bedside review: Contrast enhanced ultrasonography - a promising technique to assess renal perfusion in the ICU. Crit Care.

[R167200426977147] Perren A, Markmann M, Merlani G, Marone C, Merlani P (2011). Fluid balance in critically ill patients. Should we really rely on it?. Minerva Anesthesiol.

[R167200426977136] Flentje K M, Knight C L, Stromfeldt I, Chakrabarti A, Friedman N D (2018). Recording patient bodyweight in hospitals: are we doing well enough?. Intern Med J.

[R167200426977105] Cook D J, Simel D L (1996). The rational clinical examination. Does this patient have abnormal central venous pressure?. JAMA.

[R167200426977118] Repessé X, Charron C, Vieillard-Baron A (2013). Evaluation of left ventricular systolic function revisited in septic shock. Crit Care.

[R167200426977133] Alberti C, Brun-Buisson C, Chevret S, Antonelli M, Goodman S V, Martin C (2005). Systemic inflammatory response and progression to severe sepsis in critically ill infected patients. Am J Respir Crit Care Med.

[R167200426977140] Goldman J H, Schiller N B, Lim D C, Redberg R F, Foster E (2001). Usefulness of stroke distance by echocardiography as a surrogate marker of cardiac output that is independent of gender and size in a normal population. Am J Cardiol.

[R167200426977122] Faubel S, Patel N U, Lockhart M E, Cadnapaphornchai M A  (2014). Use of Ultrasonography in Patients with AKI. CJASN.

[R167200426977092] Quaia E, Correas J M, Mehta M, Murchison J T, Gennari A G, Beek E J R van (2018). Gary Scale Ultrasound, Color Doppler Ultrasound, and Contrast-Enhanced Ultrasound in Renal Parenchymal Diseases. Ultrasound Q.

[R167200426977089] Lerolle N, Guérot E, Faisy C, Bornstain C, Diehl J L, Fagon J Y (2006). Renal failure in septic shock: Predictive value of Doppler-based renal arterial resistive index. Intensive Care Med.

[R167200426977144] Schnell D, Deruddre S, Harrois A, Pottecher J, Cosson C, Adoui N, Benhamou D, Vicaut E, Azoulay E, Duranteau J (2012). Renal resistive index better predicts the occurrence of acute kidney injury than cystatin C. Shock.

[R167200426977128] Bertolotto M, Quaia E, Rimondini A, Lubin E, Mucelli R Pozzi (2001). Current role of color Doppler ultrasound in acute renal failure. La Radiologia Medica.

[R167200426977131] Platt J F, Rubin J M, Ellis J H (1991). Acute renal failure: possible role of duplex Doppler US in distinction between acute prerenal failure and acute tubular necrosis. Radiology.

[R167200426977096] Gheisari A, Haghighi M (2006). Diagnostic value of Doppler ultrasound in differentiating prerenal azotemia from acute tubular necrosis in children. Saudi J Kidney Dis Transpl.

[R167200426977111] Izumi M, Sugiura T, Nakamura H, Nagatoya K, Imai E, Hori M (2000). Differential diagnosis of prerenal azotemia from acute tubular necrosis and prediction of recovery by Doppler ultrasound. Am J Kidney Dis.

[R167200426977108] Mostbeck G H, Gössinger H D, Mallek R, Siostrzonek P, Schneider B, Tscholakoff D (1990). Effect of heart rate on Doppler measurements of resistive index in renal arteries. Radiology.

[R167200426977093] Umgelter A, Reindl W, Franzen M, Lenhardt C, Huber W, Schmid R M (2009). Renal resistive index and renal function before and after paracentesis in patients with hepatorenal syndrome and tense ascites. Intensive Care Med.

[R167200426977094] Leoncini G, Martinoli C, Viazzi F, Ravera M, Parodi D, Ratto E, Vettoretti S, Tomolillo C, Derchi L E, Deferrari G, Pontremoli R (2002). Changes in renal resistive index and urinary albumin excretion in hypertensive patients under long-term treatment with lisinopril or nifedipine GITS. Nephron.

[R167200426977130] Theilig D C, Münzfeld H, Auer T A, Feldhaus F, Krüger A, Dürr M, Geisel D (2020). The Renal Resistive Index in Allografts: Is Sonographic Assessment Sufficiently Reproducible in a Routine Clinical Setting? - Reproducibility of the Renal Resistive Index. Rofo.

[R167200426977124] Renberg M, Kilhamn N, Lund K, Hertzberg D, Rimes-Stigare C, Bell M (2020). Feasibility of renal resistive index measurements performed by an intermediate and novice sonographer in a volunteer population. Ultrasound J.

[R167200426977145] Quaia E (2011). Assessment of tissue perfusion by contrast-enhanced ultrasound. Eur Radiol.

[R167200426977099] Lima A, Rooij T Van, Ergin B, Sorelli M, Ince Y, Specht P A C (2018). Dynamic Contrast-Enhanced Ultrasound Identifies Microcirculatory Alterations in Sepsis-Induced Acute Kidney Injury. Crit Care Med.

[R167200426977101] Mehta R L, Pascual M T, Soroko S (2004). Spectrum of acute renal failure in the intensive care unit: the PICARD experience. Kidney Int.

[R167200426977114] Liaño F, Junco E, Pascual J, Madero R, Verde E (1998). The spectrum of acute renal failure in the intensive care unit compared with that seen in other settings. The Madrid Acute Renal Failure Study Group. Kidney Int Suppl.

[R167200426977115] Spital A, Valvo J R, Segal A J (1988). Nondilated obstructive uropathy. Urology.

[R167200426977107] Koratala A, Bhattacharya D, Kazory A (2019). Point of care renal ultrasonography for the busy nephrologist: A pictorial review. World J Nephrol.

[R167200426977090] Malbrain M, Langer T, Annane D, Gattinoni L, Elbers P, Hahn R, Laet I De, Minini A, Wong A, Ince C, Muckart D, Mythen M, Caironi P, Regenmortal N Van (2020). Intravenous fluid therapy in the perioperative and critical care setting: Executive summary of the International Fluid Academy (IFA). Ann Intensive Care.

[R167200426977125] Marik P E, Baram M, Vahid B (2008). Does central venous pressure predict fluid responsiveness? A systematic review of the literature and the tale of seven mares. Chest.

[R167200426977103] Biais M, Nouette-Gaulain K, Roullet S, Quinart A, Revel P, Sztark F (2009). A comparison of stroke volume variation measured by vigileo/ flotrac system and aortic doppler echocardiography. Anesthesia and Analgesia.

[R167200426977129] Charron C, Fessenmeyer C, Cosson C, Mazoit J X, Hebert J L, Benhamou D, Edouard A R (2006). The influence of tidal volume on the dynamic variables of fluid responsiveness in critically ill patients. Anesth Analg.

[R167200426977137] Muller L, Toumi M, Bousquet P J, Riu-Poulenc B, Louart G, Candela D, Zoric L, Suehs C, Coussaye J E De La, Molinari N, Lefrant J Y, Group AzuRéa (2011). An increase in aortic blood flow after an infusion of 100 ml colloid over 1 minute can predict fluid responsiveness: the mini-fluid challenge study. Anesthesiology.

[R167200426977148] Monnet X, Teboul J L (2008). Passive leg raising. Intensive Care Med.

[R167200426977091] Monnet X, Rienzo M, Osman D, Anguel N, Richard C, Pinsky M R, Teboul J L (2006). Passive leg raising predicts fluid responsiveness in the critically ill. Crit Care Med.

[R167200426977106] Caille V, Jabot J, Belliard G, Charron C, Jardin F, Vieillard-Baron A (2008). Hemodynamic effects of passive leg raising: An echocardiographic study in patients with shock. Intensive Care Med.

[R167200426977142] Wallace D J, Allison M, Stone M B (2010). Inferior vena cava percentage collapse during respiration is affected by the sampling location: an ultrasound study in healthy volunteers. Acad Emerg Med.

[R167200426977113] Blehar D J, Resop D, Chin B, Dayno M, Gaspari R (2012). Inferior vena cava displacement during respirophasic ultrasound imaging. Crit Ultrasound J.

[R167200426977117] Barbier C, Loubieres Y, Schmit C, Hayon J, Ricome J-L, Jardin F, Vieillard-Baron A (2004). Respiratory changes in inferior vena cava diameter are helpful in predicting fluid responsiveness in ventilated septic patients. Inten Car Med.

[R167200426977141] Feissel M, Michard F, Faller J-P, Teboul J-L (2004). The respiratory variation in inferior vena cava diameter as a guide to fluid therapy. Inten Car Med.

[R167200426977134] Bodson L, Vieillard-Baron A (2012). Respiratory variation in inferior vena cava diameter: surrogate of central venous pressure or parameter of fluid responsiveness? Let the physiology reply. Crit Care.

[R167200426977112] Li D K, Wang X T, Liu D W (2017). Association between elevated central venous pressure and outcomes in critically ill patients. Ann Intensive Care.

[R167200426977126] Vincent J L, Sakr Y, Sprung C L (2006). Sepsis in European intensive care units: results of the SOAP study. Crit Care Med.

[R167200426977146] Acheampong A, Vincent J L (2015). A positive fluid balance is an independent prognostic factor in patients with sepsis. Crit Care.

[R167200426977109] Sakr Y, Birri Rubatto, Kotfis P N (2017). Higher Fluid Balance Increases the Risk of Death From Sepsis: Results From a Large International Audit. Crit Care Med.

[R167200426977123] Jozwiak M, Silva S, Persichini R (2013). Extravascular lung water is an independent prognostic factor in patients with acute respiratory distress syndrome. Crit Care Med.

[R167200426977120] Ryan T, Petrovic O, Dillon J C, Feigenbaum H, Conley M J, Armstrong W F (1985). An echocardiographic index for separation of right ventricular volume and pressure overload. J Am Coll Cardiol.

[R167200426977127] Bleeker G B, Steendijk P, Holman E R (2006). Acquired right ventricular dysfunction. Heart.

[R167200426977139] Greenstein Y Y, Mayo P H (2018). Evaluation of Left Ventricular Diastolic Function by the Intensivist. Chest.

[R167200426977143] Lanspa M J, Gutsche A R, Wilson E L (2016). Application of a simplified definition of diastolic function in severe sepsis and septic shock. Crit Care.

[R167200426977119] Nagueh S F, Appleton C P, Gillebert T C, Marino P N, Oh J K, Smiseth O A, Waggoner A D, Flachskampf F A, Pellikka P A, Evangelista A (2009). Recommendations for the evaluation of left ventricular diastolic function by echocardiography. J Am Soc Echocardiogr.

[R167200426977097] Öhman J, Harjola V P, Karjalainen P, Lassus J (2019). Rapid cardiothoracic ultrasound protocol for diagnosis of acute heart failure in the emergency department. Eur J Emerg Med.

[R167200426977095] Lichtenstein D A, Mezière G A, Lagoueyte J F, Biderman P, Goldstein I, Gepner A (2009). A-lines and B-lines: lung ultrasound as a bedside tool for predicting pulmonary artery occlusion pressure in the critically ill. Chest.

[R167200426977121] Lichtenstein D, Karakitsos D (2012). Integrating lung ultrasound in the hemodynamic evaluation of acute circulatory failure (the fluid administration limited by lung sonography protocol). J Crit Care.

[R167200426977110] Lichtenstein D, Mézière G, Biderman P, Gepner A, Barré O (1997). The comet-tail artifact. An ultrasound sign of alveolar-interstitial syndrome. Am J Respir Crit Care Med.

[R167200426977104] Hubert Arnaud, Girerd Nicolas, Hervé Le Breton Elena, Galli Ichraq, Latar Maxime, Fournet Philippe, Mabo Frederic, Schnell Christophe, Leclercq Erwan, Donal (2019). Diagnostic accuracy of lung ultrasound for identification of elevated left ventricular filling pressure. International Journal of Cardiology.

[R167200426977138] Beaubien-Souligny W, Rola P, Haycock K, Bouchard J, Lamarche Y, Spiegel R, Denault A Y (2009). Quantifying systemic congestion with Point-Of-Care ultrasound: development of the venous excess ultrasound grading system. Ultrasound J.

[R167200426977100] Beaubien-Souligny W, Bouchard J, Desjardins G, Lamarche Y, Liszkowski M, Robillard P, Denault A (2017). Extracardiac signs of fluid overload in the critically ill cardiac patient: a focused evaluation using bedside ultrasound. Can J Cardiol.

[R167200426977116] Tang W H, Kitai T (2016). Intrarenal venous flow: a window into the congestive kidney failure phenotype of heart failure?. JACC Heart Fail.

[R167200426977098] Beaubien-Souligny W, Benkreira A, Robillard P, Bouabdallaoui N, Chassé M, Desjardins G, Lamarche Y, White M, Bouchard J, Denault A (2018). Alterations in Portal Vein Flow and Intrarenal Venous Flow Are Associated With Acute Kidney Injury After Cardiac Surgery: A Prospective Observational Cohort Study. J Am Heart Assoc.

